# Impact of dermatology targeted immunotherapies on autoimmune thyroid diseases: cytokine-network crosstalk and thyroid safety

**DOI:** 10.3389/fimmu.2026.1890822

**Published:** 2026-07-27

**Authors:** Jiahao Zhou, Ruiyang Han, Tianshu Gao

**Affiliations:** 1 The First Clinical College, Liaoning University of Traditional Chinese Medicine, Shenyang, Liaoning, China; 2 Department of Endocrinology, Affiliated Hospital of Liaoning University of Traditional Chinese Medicine, Shenyang, Liaoning, China

**Keywords:** autoimmune thyroid disease, dermatological diseases, graves’ disease, hashimoto’s thyroiditis, targeted immunotherapies

## Abstract

Autoimmune thyroid diseases (AITDs), including Hashimoto’s thyroiditis (HT) and Graves’ disease (GD), affect approximately 2–5% of the global population. They are the most common organ-specific autoimmune disorders worldwide. Growing epidemiological and immunological evidence indicates that AITDs frequently coexist with immune-mediated dermatological diseases. These include atopic dermatitis (AD), psoriasis, alopecia areata (AA), vitiligo, and chronic spontaneous urticaria (CSU). Targeted immunotherapies have become central to the treatment of many immune-mediated skin diseases. Although these therapies are designed to act on specific immune pathways, they may also exert broader systemic immunomodulatory effects. However, thyroid function and thyroid autoantibodies are not routinely assessed in patients with immune-mediated dermatological conditions. This is also true for some patients with known or suspected AITDs. In this narrative review, we summarize the immunological overlap between cutaneous inflammation and autoimmune thyroid disease. We also discuss thyroid-related evidence for IL-4Rα inhibitors, IL-17 inhibitors, JAK inhibitors, IL-12/23 inhibitors, and immune checkpoint inhibitors (ICIs). Current evidence suggests that ICIs have the strongest established association with thyroid dysfunction. In contrast, the thyroid-related effects of dupilumab and ustekinumab are supported mainly by rare case reports. IL-17 inhibitors and selective IL-23p19 inhibitors may theoretically attenuate thyroid inflammatory pathways. However, this possibility has not yet been confirmed in clinical studies. Based on the available evidence, we propose a risk-stratified approach to thyroid surveillance across targeted immunotherapies. Routine thyroid monitoring is most strongly supported for ICIs. For patients at increased thyroid risk, selective assessment of thyroid function and thyroid autoantibodies may also be considered during treatment with dupilumab or ustekinumab. For oral JAK inhibitors, monitoring may be particularly relevant before treatment, during therapy, and after discontinuation because of the potential for immune rebound. Prospective studies are needed to define which patient subgroups would benefit most from thyroid surveillance during targeted immunotherapy.

## 1 Introduction

Hashimoto’s thyroiditis (HT) is a chronic autoimmune thyroid disease (AITDs). In iodine-sufficient regions, it is the main cause of hypothyroidism (1). It is characterized by diffuse lymphocytic infiltration of the thyroid gland, progressive follicular destruction, and the production of thyroid-specific autoantibodies, most notably anti-thyroid peroxidase antibodies (TPOAb) and anti-thyroglobulin antibodies (TgAb). HT affects approximately 5-10% of the global population, with a striking female predominance of approximately 4:1 in adults, and its prevalence increases with age (1, 2).

Graves’ disease (GD) is the most common cause of hyperthyroidism. The peak age of onset is between 30 and 50 years, and the prevalence is significantly higher in women than in men. The reported prevalence in China is approximately 2.0–3.0%, whereas that in Western countries is about 0.5–2.0%. The peak incidence is concentrated between 30 and 50 years of age, although GD can occur at any age (3).

Crucially, AITDs do not occur in immunological isolation. Epidemiological studies have consistently shown that HT frequently coexists with a range of immune-mediated diseases. These include type 1 diabetes mellitus, rheumatoid arthritis, systemic lupus erythematosus, coeliac disease, and an increasing number of immune-mediated dermatological disorders (1, 2). Patients with AD have a tendency towards an increased risk of HT and GD. This association may be more pronounced in children or younger populations (4, 5). In patients with AA, a meta-analysis of thyroid function and thyroid autoantibody screening showed higher rates of thyroid dysfunction, thyroid autoantibody positivity, and AITDs than those observed in the general population (6). National population-based studies from South Korea and Israel have also reported that individuals with AA are more likely to have HT or GD (7, 8).Among immune-mediated skin diseases, vitiligo has shown one of the most consistent epidemiological associations with AITD, particularly HT (9). Evidence also supports an association with GD, although the prevalence of GD in patients with vitiligo is generally lower than that of HT (10). Similarly, several studies have reported a higher prevalence of AITDs in patients with psoriasis than in controls. Psoriasis has also been associated with TPOAb positivity, hypothyroidism, and hyperthyroidism (11, 12). CSU is another condition closely linked to thyroid autoimmunity. Patients with AITDs have an increased risk of developing CSU. Conversely, patients with CSU have a markedly higher risk of TPOAb positivity, approximately five times that of the general population (13, 14). Compared with the five immune-mediated skin diseases discussed above, evidence for a natural comorbid relationship between cutaneous melanoma and AITDs remains limited. A nationwide population-based study from South Korea reported a higher prevalence of HT among patients with melanoma (15). However, a retrospective matched cohort study from Mayo/Olmsted County did not show an increased risk of melanoma in patients with HT (16). Similarly, no significant increase in melanoma risk has been observed in patients with GD (17). Notably, immune checkpoint inhibitors, which are widely used in melanoma treatment, can induce immune-related thyroid dysfunction (18). For this reason, melanoma-related thyroid events in the context of immunotherapy are also discussed in this review.

The shared immunological mechanisms underlying AITDs and immune-mediated skin diseases have been widely investigated (19–22). However, it remains unclear whether therapies targeting one immune-mediated disorder may affect the onset, progression, or activity of another.

This review focuses on targeted immunomodulatory therapies used for immune-mediated dermatological diseases that have been reported to coexist with AITDs. These diseases include atopic dermatitis, psoriasis, alopecia areata, vitiligo, and chronic spontaneous urticaria. Although melanoma is not classified as an immune-mediated skin disease, it is included because immune checkpoint inhibitors can affect thyroid function.

The therapeutic classes discussed in this review include IL-4Rα inhibitors, IL-17 inhibitors, JAK inhibitors, immune checkpoint inhibitors, and IL-12/23 inhibitors. This review does not seek to establish a direct causal relationship between targeted immunomodulatory therapies and AITDs. Instead, it addresses two major questions. First, it summarizes the shared immune pathways that link dermatological targeted immunosuppressants with AITDs. Second, it discusses how different classes of targeted therapies used in dermatology may influence thyroid autoimmunity through their distinct immunological effects.

## 2 Methods

We conducted a literature search of PubMed, Embase, and Web of Science for articles published from database inception through April 1st. Search terms including autoimmune thyroid disease, Hashimoto thyroiditis, Graves disease, thyroid autoimmunity, thyroid peroxidase antibody, thyroglobulin antibody, TSH receptor antibody, atopic dermatitis, psoriasis, alopecia areata, vitiligo, and chronic spontaneous urticaria, combined with inhibitor class terms including IL-4Rα inhibitor, IL-17 inhibitor, JAK inhibitor, IL-12/23 inhibitor, immune checkpoint inhibitor.

Eligible study types included case reports, case series, cohort studies, randomized controlled trials, post-marketing pharmacovigilance analyses, meta-analyses, and mechanistic or basic science studies addressing thyroid function, thyroid autoantibody status, or thyroid-related adverse events in the context of the five drug classes under review. Only studies published in English were included. Given the narrative design of this review, formal systematic methodology including PRISMA reporting and structured risk of bias assessment was not applied.

## 3 The core immune network in AITDs and its therapeutic relevance to dermatological targeted therapies

### 3.1 Common genetic susceptibility

GD and HT are two major clinical phenotypes within the AITDs spectrum and share a high degree of genetic overlap in disease susceptibility. In addition to MHC class II genes, several non-HLA susceptibility loci have been associated with AITDs, including immune-regulatory genes such as CD40, CTLA-4, PTPN22, FOXP3, and CD25, as well as thyroid-specific genes such as thyroglobulin and TSHR (23). GD and HT share multiple susceptibility loci, including HLA, PTPN22, and CTLA-4, whereas the association with TSHR appears to be more specific to GD (24). A study of monozygotic twins have reported that concordance for thyroid antibody positivity may reach 80%, suggesting that both genetic and environmental factors contribute to disease development (25). Members of the same family may present with HT, GD, or features of both diseases, further supporting a shared genetic background between these two AITDs phenotypes (26).

### 3.2 Th17/Treg axis and IL-17 blockade

Disruption of the Th17/Treg balance represents one of the central immunological mechanisms shared by GD and HT. A meta-analysis of a cohort of AITDs indicates that a Th17/Thg cell imbalance exists in newly diagnosed HT and GD patients (27).

The Th17/Treg imbalance in GD reflects a multilayered regulatory network encompassing genetic predisposition, cytokine-driven immune skewing, and metabolic reprogramming. At the genetic level, PTPN22 can regulate the balance between Th17 and Treg by down-regulating the Lck/Zap70 signaling axis (28).The polymorphisms of the CD25 gene and the VDR gene contribute to the development of GD by increasing the ratio of Th17/Treg cells (3). In GD patients, the low level of NR4A2 may inhibit the differentiation of Tregs, but has no effect on the differentiation of Th17 cells (29). Epigenetic mechanisms compound these genetic vulnerabilities. Upregulation of miR-142 suppresses Treg programming (30), while WTAP-mediated N6-methyladenosine modification of THBS1 drives promoting glycolysis in CD4 T cells and affect the balance between Th17 and Treg cells (31). Furthermore, miR-23a-3p and lncRNA-MEG3 also contribute to the imbalance between Th17 and Treg cells, thereby participating in the progression of GD (3). Th17 cells preferentially utilize aerobic glycolysis rather than the fatty acid oxidation that sustains Treg function, thereby establishing a metabolic environment that intrinsically favors Th17 persistence within the inflammatory milieu of GD (32).

At the cytokine level, the pro-inflammatory microenvironment of newly diagnosed GD patients is characterized by elevated IL-6 and TGF-β (33, 34), which cooperatively regulate RORγt and Foxp3 (35), regulating the Th17/Treg cell axis (36, 37). Hayashi et al. conducted a classic study examining the −31C/T polymorphism in the promoter region of the IL1B gene. The −31T allele, which is associated with high IL-1β production, occurred at a significantly higher frequency in patients with intractable GD than in patients with GD in remission. The same study showed that the proportion of IL-17 positive Th17 cells in peripheral blood was higher in patients carrying the T allele. IL-1β is the cytokine that drives the differentiation and proliferation of Th17 cells (38). IL-21, secreted by Th17 cells in an autocrine manner, sustains Th17 expansion and further impairs Treg suppressive capacity, by inducing CD4T cells to differentiate into Th17 cells (39). Comprehensive immunophenotypic analyses have confirmed significantly elevated circulating Th17 cells in GD patients (40). The disruption of immune tolerance in GD extends beyond T cells. CD19+CD1d-hi CD5+ regulatory B cells are simultaneously depleted, indicating a broad collapse of suppressive immune networks rather than an isolated T cell defect (41). Treg insufficiency is further evidenced by absolute reductions in peripheral Treg numbers, as quantified by flow cytometry in clinical cohorts (42). In addition, gut microbiota dysbiosis may disturb the Th17/Treg balance and participate in GD pathogenesis (3).

The Th17/Treg imbalance observed in HT is shaped by the convergence of cytokine-mediated signaling, transcriptional dysregulation, epigenetic modification, and metabolic reprogramming. At the cytokine level, serum concentrations of IL-6 and IL-23 are markedly elevated in patients with HT (43), and these two cytokines act in concert to phosphorylate and activate STAT3, which in turn upregulates RORγt transcription and simultaneously relieves the inhibitory constraint that FOXP3 exerts on RORγt, thereby steering naive CD4^+^ T cells toward the Th17 lineage (44). In parallel, the anti-inflammatory cytokines TGF-β and IL-10 are significantly reduced in the circulation of HT patients (45). A study performed immunohistochemistry on thyroid tissue obtained after thyroidectomy from 29 patients with AITDs, comprising 21 patients with HT and 8 patients with GD, together with 18 patients with colloid goiter serving as controls. Immunopositivity for IL-23 and IL-1β was significantly higher in the HT group than in both the GD group and the colloid goiter control group. No significant difference was observed between the GD group and the control group. These findings suggest that IL-1β and IL-23, two pathogenic Th17 inducing cytokines, may participate in the pathogenesis of HT (46). The resulting contraction of the Treg compartment, together with its functional impairment, sustains elevated serum IL-17A levels (47).

At the transcriptional level, RORγt mRNA is significantly overexpressed in peripheral blood mononuclear cells of HT patients, whereas FOXP3 mRNA is correspondingly downregulated (48). The protective transcription factor BACH2, whose expression sustains FOXP3 programme stability, is also functionally compromised in this context (49). The broader imbalance across T cell-related transcription factor ratios, including RORα/FOXP3 and T-bet/FOXP3, has been corroborated in HT cohorts (50).

At the epigenetic level, miR-146a is abnormally upregulated in the peripheral blood of HT patients and positively correlates with IL-6, IL-23, and IL-17 while inversely correlating with IL-10, implicating this microRNA as an upstream modulator of the pro-Th17 cytokine network (51). Aberrant miR-142 expression has similarly been linked to Th17/Treg imbalance in pediatric patients with AITDs (30).

**Figure 1:**
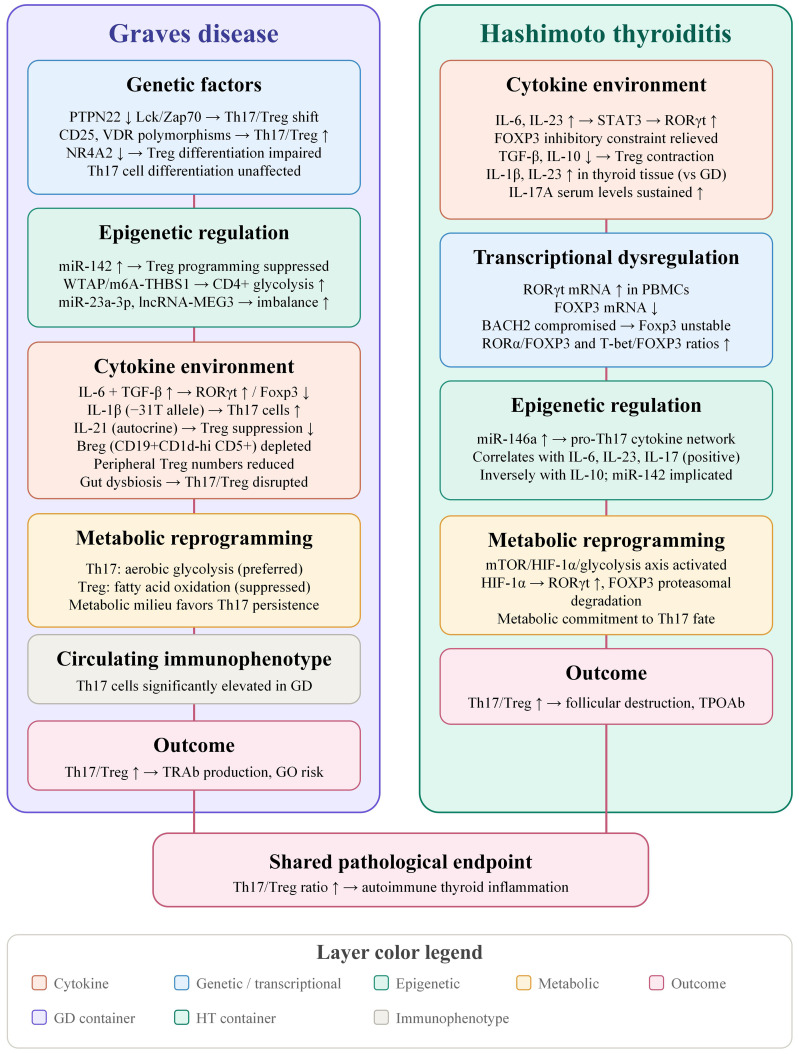
Th17/Treg dysregulation in AITDs. ↓ means a decrease, ↑ means an increase.

At the metabolic level, the mTOR/HIF-1α/glycolysis axis is abnormally activated in CD4^+^ T cells of HT patients (52). HIF-1α, acting downstream of mTOR, selectively promotes RORγt expression and targets FOXP3 protein for proteasomal degradation, thereby consolidating commitment to the Th17 fate (53). In conclusion, these multilayered regulatory perturbations reinforce one another and constitute the immunological substrate for the sustained pro-inflammatory shift of the Th17/Treg axis that characterizes HT. This cellular plasticity provides an important biological basis for Th17/Treg imbalance in AITDs (**Figure 1**).

This axis is directly relevant to IL-17 inhibitors used in dermatology (54). IL-17A blockade, or broader inhibition of IL-17A/F signaling, can suppress downstream inflammatory mediators, chemokine production, and neutrophil recruitment in immune-mediated skin diseases (54–56). From the perspective of AITDs, IL-17 inhibition may theoretically attenuate Th17-driven thyroid inflammation, particularly in HT (57, 58). A concrete illustration comes from a murine checkpoint-inhibitor thyroiditis model in which anti-IL-17A antibody treatment significantly reduced autoimmune thyroid infiltration without compromising anti-tumor efficacy (133). In addition, there are some case reports indicating that IL-17 inhibitors can improve Graves’ ophthalmopathy, and subacute thyroiditis (59, 60). However, whether IL-17 blockade can meaningfully alter thyroid autoimmunity or thyroid function in patients with AITDs remains insufficiently established (57). Therefore, IL-17 inhibitors should be discussed as agents with a plausible thyroid-relevant mechanism, rather than as confirmed therapeutic modulators of AITDs (61) (**Figure 1**).

### 3.3 JAK–STAT signaling axis and JAK inhibition

The JAK–STAT pathway represents a central cytokine-transduction hub linking extracellular inflammatory signals with transcriptional programs that regulate T-cell differentiation, B-cell activation, innate immune responses (62). Multiple cytokines implicated in HT and GD converge on distinct JAK–STAT modules.

The JAK-STAT pathway mainly participates in the pathogenesis of HT in three aspects. IFN-γ predominantly activates the JAK1/JAK2–STAT1 pathway and promotes Th1-associated inflammation, antigen presentation, and tissue-destructive immune responses (63, 64). IL-12 acts through JAK2/TYK2-STAT4, promoting the differentiation of Th1 cells and further enhancing the production of IFN-γ, thereby amplifying the cellular immune damage in HT (65). IL-6 signals via JAK1/JAK2/TYK2–STAT3 and is essential for the initial induction of Th17 differentiation from naïve precursors, whereas IL-23 acts primarily through TYK2/JAK2–STAT3 and is required for the subsequent stabilization and pathogenic programming of committed Th17 cells rather than their *de novo* generation (66, 67).

In GD, IL-4 and IL-13 signal mainly through JAK1/JAK3 and STAT6, thereby supporting Th2-associated humoral immunity and B-cell responses (68, 69). IL-21 can promote the Tfh-B cell response and TRAb production through the JAK1-JAK3/STAT3 axis. Furthermore, IL-6 can also promote Tfh and Th17-related immune responses through JAK/STAT3, and indirectly facilitate B cell activation and the formation of autoantibodies (70).

Therefore, JAK–STAT signaling provides a mechanistic bridge between cytokine-network imbalance and the Th1/Th2 and Th17/Treg immune deviations observed in HT and GD. This axis is particularly important for the present review because oral JAK inhibitors are increasingly used in immune-mediated dermatological diseases, including AD, AA, vitiligo, and psoriasis-related indications (71, 72).

### 3.4 Th1/Th2 axis and IL-4Rα blockade

GD and HT have often been described through the Th1/Th2 paradigm. In this traditional model, GD is regarded as a predominantly antibody-mediated disease with a Th2-associated profile, whereas HT is viewed as a Th1-skewed disorder characterized by lymphocytic thyroid infiltration and follicular destruction. However, this binary model is now considered overly simplified. AITDs are shaped by multiple immune pathways.

The IgG subclass distribution of thyroid autoantibodies provides indirect evidence of cytokine bias, but it does not define the full immune phenotype of AITDs. Th1-associated cytokines, such as IFN-γ, can promote IgG1 subclass production, whereas Th2-associated cytokines, such as IL-4, can favor IgG4 subclass production (73). In early GD, TRAb is mainly of the IgG1 subclass, suggesting that Th1-associated cytokine signals may also contribute to GD pathogenesis (74). Therefore, GD should not be regarded as a purely Th2-mediated disease.

A similar complexity is observed in HT. TPOAb in HT may include both IgG1 and IgG4 subclasses. This finding suggests that Th2-associated pathways may also participate in selected HT phenotypes, especially in IgG4-related or IgG4-enriched forms of HT (75, 76). Thus, GD and HT should be understood as overlapping autoimmune conditions with different dominant effector patterns. Their differences are more closely related to downstream immune effects, such as stimulatory autoantibody production in GD and destructive thyroidal immune injury in HT, rather than to the exclusive dominance of one T-helper cell subset.

This more nuanced view is relevant to IL-4Rα inhibitors such as dupilumab. Dupilumab blocks IL-4 and IL-13 signaling and reduces type 2 cytokine-driven immune responses in immune-mediated dermatological diseases such as atopic dermatitis. Cases of vitiligo, psoriasis, and seronegative arthritis have been reported during dupilumab treatment (77–79). These events suggest that IL-4Rα blockade may alter immune balance in some susceptible individuals. However, the immune consequences are likely to depend on disease context, host susceptibility, baseline cytokine networks, and tissue-specific immune regulation.

In relation to AITDs, IL-4Rα inhibition should not be interpreted as causing a simple shift from Th2 to Th1 immunity (80). A more cautious interpretation is that blockade of IL-4 and IL-13 may reshape the balance among T-cell subsets, B-cell responses, and autoantibody-related pathways in predisposed individuals. This may be relevant to rare reports of thyroid dysfunction or thyroid autoimmunity during IL-4Rα inhibitor therapy (80–82). At present, this association remains a mechanistically plausible hypothesis supported mainly by limited clinical observations. It should not be considered established causality.

### 3.5 IL-12/23 axis and the interface between Th1 and Th17 immunity

The IL-12/23 axis occupies an important upstream position in the coordination of Th1 and Th17 immunity. IL-12 promotes Th1 differentiation and IFN-γ production, whereas IL-23 supports the expansion, stabilization, and pathogenicity of Th17 cells (83). Because both Th1-associated tissue-destructive inflammation and Th17/Treg imbalance are involved in AITDs, the IL-12/23 axis provides another mechanistic interface between thyroid autoimmunity and dermatological targeted therapy.

IL-12/23 inhibitors, represented by ustekinumab, target the shared p40 subunit of IL-12 and IL-23, thereby suppressing both Th1- and Th17-related immune pathways (84, 85). In psoriasis and related immune-mediated skin diseases, this mechanism reduces inflammatory cytokine amplification and pathogenic T-cell responses (86). From an AITDs perspective, blockade of IL-12/23 signaling could theoretically attenuate Th1- and Th17-associated thyroid inflammation. However, because IL-12 and IL-23 also participate in broader immune homeostasis, the net effect on thyroid autoimmunity is difficult to predict (87, 88). Current evidence linking IL-12/23 inhibition to thyroid dysfunction remains limited, and reported associations should be interpreted cautiously.

Selective IL-23p19 inhibitors differ from IL-12/23p40 inhibitors because they suppress IL-23-driven Th17 responses while sparing IL-12-mediated Th1 immunity (89). This more selective mechanism may theoretically reduce Th17-related inflammatory amplification without broadly inhibiting Th1 responses. However, whether selective IL-23 blockade has clinically meaningful effects on HT, GD, or thyroid autoantibody dynamics remains unknown. Therefore, both IL-12/23 and IL-23 inhibitors should be framed as biologically relevant to AITDs immune networks but not yet clinically validated as modifiers of thyroid autoimmunity.

## 4 Dermatological targeted therapies and the impact of thyroid autoimmunity

The past decade has witnessed a therapeutic revolution in dermatology, with dermatological targeted therapies transforming the management of moderate-to-severe AD, psoriasis, AA, and melanoma. These agents, including IL-4Rα inhibitors, IL-17 inhibitors, JAK inhibitors, IL-23/IL-12 inhibitors, and immune ICIs achieving their therapeutic effects through precise cytokine or immune checkpoint blockade. Their efficacy and systemic immunological reach are well established. However, a critical dimension has been systematically overlooked in clinical practice and regulatory frameworks is that the impact of these agents on AITDs.

Thyroid function monitoring is not a standard component of pre-treatment screening or follow-up protocols for most dermatological biologics, with the partial exception of ICIs (90). Yet an accumulating body of case reports and mechanistic studies suggests that several of these agents can trigger or exacerbate AITDs. The mechanisms are diverse. Some agents cause immune drift that amplifies pathological Th1 or Th17 responses in the thyroid. Others remove inhibitory checkpoints that normally restrain autoreactive T cells or suppressing the very cytokines that drive thyroid destruction.

### 4.1 Immune checkpoint inhibitors

#### 4.1.1 Mechanism of action and dermatological indication

ICIs are used to treat melanoma and include PD-1, PD-L1 and CTLA-4 inhibitors. It is the only dermatological biologic agent that has included thyroid function as part of the routine baseline monitoring and during the treatment period.

CTLA-4 is primarily expressed on T cells, including activated and regulatory T cells, and plays a role in the initial phase of the immune response in lymphoid organs. It bypasses the co-stimulatory receptor CD28 and attenuates the initial activation of T cells by competitively binding to the B7 ligands (CD80/CD86) on antigen-presenting cells (91). CTLA-4 inhibitor lowers the threshold for T-cell activation and expands the population of melanoma-reactive CD8^+^ T cells. It also depletes tumor-infiltrating Tregs through antibody-dependent cell-mediated cytotoxicity and can reshape memory T cell subset composition. The CTLA-4 and PD-1/PD-L1 pathways are intricately interwoven. CTLA-4 inhibition promotes T cell activation and IFN-γ secretion, which upregulates PD-L1 expression on tumor cells and weakens the efficacy of CTLA-4 monotherapy. Dual blockade can prevent this adaptive resistance, increase the CD8^+^/Treg ratio, and significantly enhance the therapeutic effect (92). Combined therapy produced a 5-year overall survival rate of 52%, which was significantly higher than the rates for PD-1 monotherapy (44%) and CTLA-4 monotherapy (26%) (93). Inhibiting CTLA-4 promotes the initial activation of T cells and increases the breadth and diversity of responses, whereas inhibiting PD-1 maintains the functional state of effector T cells during the response stage (94).

#### 4.1.2 Clinical evidence

Thyroid irAE is primarily triggered by antibodies that target PD-1 and PD-L1. CTLA-4 inhibitors rarely cause it on their own. The typical clinical course involves a two-phase change: transient thyrotoxicosis usually occurs within 2–6 weeks, followed by persistent hypothyroidism (95). The incidence of thyroid irAE was highest in the combination therapy group and lowest in the CTLA-4 monotherapy group. This suggests that differences in the mechanisms of action of ICIs are directly related to varying irAE incidence rates (96). In addition, combined ICI therapy (PD-1+CTLA-4) leads to a faster progression of thyroiditis, resulting in a higher proportion of symptomatic patients. Several studies have reported an exacerbation of pre-existing HT and subclinical hypothyroidism in patients undergoing immunotherapy, indicating that autoimmune and inflammatory mechanisms may exert a synergistic destructive effect (97) (**Table 1**).

**Table 1:** Immune checkpoint inhibitors with reported thyroid immune-related adverse events.

Agent (brand name)	Mechanism class	IgG Subclass/Fc features	Reported thyroid irAEs	Key references
Anti-PD-1 Agents				
Pembrolizumab (Keytruda)	Anti-PD-1	IgG4	Hypothyroidism biphasic thyrotoxicosis (painless thyroiditis) Graves’ disease (rare) Hyperthyroidism	(133–136)
Nivolumab (Opdivo)	Anti-PD-1	IgG4	Hypothyroidism thyrotoxicosis painless thyroiditis Graves’ disease	(133, 134, 137, 138)
Cemiplimab (Libtayo)	Anti-PD-1	IgG4	Hypothyroidism hyperthyroidism	(137, 139)
Camrelizumab (Airuika)	Anti-PD-1	IgG4	Hypothyroidism hyperthyroidism thyroiditis	(140, 141)
Sintilimab (Tyvyt)	Anti-PD-1	IgG4	Hypothyroidism hyperthyroidism	(140–142)
Toripalimab (Tuoyi)	Anti-PD-1	IgG4	Hypothyroidism hyperthyroidism	(140, 142)
Tislelizumab (BeiGene)	Anti-PD-1	IgG4	Hypothyroidism hyperthyroidism	(140, 141)
Anti-PD-L1 Agents				
Atezolizumab (Tecentriq)	Anti-PD-L1	IgG1 (Fc-engineered; ADCC eliminated)	Hypothyroidism (predominant) thyrotoxicosis (less common)	(139, 140, 143)
Durvalumab (Imfinzi)	Anti-PD-L1	IgG1 (Fc-engineered; ADCC eliminated)	Thyroiditis hypothyroidism	(95, 133, 138, 144)
Avelumab (Bavencio)	Anti-PD-L1	IgG1 (native Fc; ADCC retained)	Hypothyroidism (predominant) thyrotoxicosis	(138, 139, 143)
Anti-CTLA-4 Agents				
Ipilimumab (Yervoy)	Anti-CTLA-4	IgG1	Hypothyroidism hyperthyroidism subclinical thyroiditis	(133, 134, 137, 145)
Tremelimumab (Imjudo)	Anti-CTLA-4	IgG2	Hypothyroidism hyperthyroidism (low frequency)	(137, 140, 145)
Combination Regimens				
Nivolumab + Ipilimumab (CheckMate)	PD-1 + CTLA-4	IgG4 + IgG1	Hypothyroidism hyperthyroidism thyroiditis Graves’ disease	(95, 133, 134, 138)
Durvalumab + Tremelimumab (HIMALAYA)	PD-L1 + CTLA-4	IgG1 + IgG2	Hypothyroidism hyperthyroidism	(133, 138, 140)

irAE, immune-related adverse event; ADCC, antibody-dependent cell-mediated cytotoxicity; Fc, fragment crystallizable region; PD-1, programmed death-1; PD-L1, programmed death-ligand 1; CTLA-4, cytotoxic T-lymphocyte-associated protein 4.

In patients with thyroid irAE, lymphocyte accumulation within the thyroid can be observed via a fine-needle aspiration biopsy, a procedure similar to HT. Genetic analysis has revealed the involvement of several genes, including CTLA-4, CBLB, PTPN22 and CD69, which are all related to immune regulation and autoimmunity (98). Lechner et al. discovered that, in patients with ICI thyroiditis, thyroid tissue mainly contains clonally expanded CD8^+^ T cells, whereas in patients with HT, CD4^+^ T cells are predominant. This difference in cellular immune composition suggests that the pathogenesis of the two conditions is not entirely the same (99). Further studies have shown that thyroiditis induced by pembrolizumab is mediated by circulating CD56/CD16 natural killer cells, which differ from the cell phenotype of AITDs. Another study described an association between PD-L1 and PD-L2 expression on the thyroid surface, destructive thyroiditis and the exacerbation of pre-existing HT and subclinical hypothyroidism in patients receiving immunotherapy, suggesting a potential synergistic effect between autoimmune and inflammatory mechanisms (97).

### 4.2 IL-4Rα inhibitors: dupilumab

#### 4.2.1 Mechanism of action and dermatological indication

Dupilumab is a fully human IgG4 monoclonal antibody that works by blocking the IL-4 and IL-13 receptor signaling pathway (100). It is used to treat allergic diseases such as atopic dermatitis, asthma, nasal polyps accompanied by chronic sinusitis and other allergic diseases (101–103). The interleukin-4 receptor α subunit (IL-4Rα) is a key target for dupilumab in its therapeutic applications. Dupilumab works by binding to IL-4Rα, which simultaneously blocks the IL-4 and IL-13 signaling pathways (104). After IL-4 and IL-13 activate the JAK1/JAK3-STAT6 pathway via IL-4Rα, they upregulate GATA3 and inhibit T-bet, thus maintaining the balance of Th1 polarization. Blocking IL-4Rα deactivates the STAT6 pathway and disrupts the balance of Th1 cells (105). As previously mentioned, HT is dominated by Th1 cells. Atopic dermatitis, on the other hand, belongs to type 2 inflammation and is dominated by Th2 cells. Since type 1 and type 2 inflammation are in a state of dynamic balance, dupilumab inhibits the IL-4 and IL-13 pathways (106). Furthermore, dupilumab is known to induce disinhibition of Th17 cells. In a report of 37,848 adverse reactions related to dupilumab, some Th17-mediated diseases showed a significant increase, such as seronegative arthritis, enthesitis/enthesopathy, psoriasis, and iridocyclitis (79). Therefore, there is also an assumption that the inhibition of the Th2-mediated pathway may exacerbate the Th1/Th17-dependent immune response (107). Th17-mediated pathways have received growing attention in the pathogenesis of both HT and GD. Although cases of dupilumab-associated HT and GD have been reported, the potential contribution of Th17 disinhibition has not been addressed in these reports. Therefore, we propose that dupilumab may promote AITDs through two distinct immunological pathways.

#### 4.2.2 Clinical evidence

A case report have described thyrotoxicosis occurring after dupilumab treatment (80). Another case report indicates that during the treatment with dupilumab, patients experienced both elevated tumor markers and HT. After discontinuation of the medication, the symptoms and marker levels gradually subsided, suggesting that dupilumab may increase the risk of HT (105). Currently, the long-term safety data is insufficient, and further research is required to investigate the link between dupilumab and autoimmune events. Asymptomatic thyroiditis is also a type of AITDs. Another study documented a 49-year-old male who received dupilumab treatment for severe atopic dermatitis. Four months later, he developed hyperthyroidism, with decreased radioactive iodine uptake in the thyroid, diffuse hypoechoic areas on ultrasound, and lymphocyte infiltration on pathology. Three weeks later, the condition progressed to hypothyroidism, which was consistent with the typical course of asymptomatic thyroiditis (108). Although Dupilumab has been reported in cases of GD, HT, and painless thyroiditis, its prescribing information does not include thyroid function as a routine baseline test item. Given the existence of relevant case reports, if the patient has a history of thyroid disease, positive thyroid antibodies, or a background of multiple autoimmune diseases, individualized testing of TSH, FT4, and thyroid autoantibodies can be considered.

### 4.3 IL-23/IL-12 inhibitors

#### 4.3.1 Mechanism of action and dermatological indication

IL-12 and IL-23 are structurally related heterodimeric cytokines that share the p40 subunit, yet differ substantially in their biological functions. IL-12/23 inhibitors target the shared IL-12/23 p40 subunit (ustekinumab) or the IL-23 p19 subunit (109, 110). IL-23 blockade selectively inhibits the pathological Th17 cell subset in psoriatic lesions via risankizumab (111). This selectivity enables effective treatment while preserving some physiological immune functions (112, 113). In the field of dermatology, they are mainly used for treating psoriasis.

Ustekinumab binds specifically to the p40 subunit shared by IL-12 and IL-23. This blocks their binding to receptors, reducing their activity and alleviating the inflammatory response. In contrast, risankizumab binds to the p19 subunit of IL-23, thereby inhibiting the release of pro-inflammatory cytokines and chemokines and reducing inflammation and psoriasis symptoms (114). Inhibitors target the shared IL-23 p19 subunit specifically binds to the IL-23p19 subunit and does not bind to IL-12 or IL-12/23p40. It only prevents the interaction between IL-23 and IL-23R, without affecting the signal transduction of IL-12 (109). The baseline monitoring for ustekinumab usually includes blood routine tests, electrolytes, liver function tests, inflammatory markers, tuberculosis screening, hepatitis B/hepatitis C/HIV screening, and vaccination status, etc. However, thyroid function is not included in the regular list. Thyroid function monitoring is an additional item for high-risk groups, rather than a mandatory baseline test for all individuals.

#### 4.3.2 Clinical evidence

The IL-23/IL-17 axis and Th17/Treg imbalance are key factors in the onset of AITDs. Serum and tissue levels of IL-17 are positively correlated with TPOAb and TGAb titers. Th17 dominance even precedes tissue damage mediated by Th1 (57). Ustekinumab inhibits the Th1/Th17-mediated pathways, which may cause a shift in the Th1/Th2 balance. The Th2-mediated pathways are believed to play a key role in the pathogenesis of GD. An increase in Th2 activity can result in an increase in autoantibodies against the TSH receptor, which stimulates hyperfunction in thyroid follicular cells. Cases of autoimmune thyroiditis have been reported following treatment with ustekinumab, with thyroid hormone levels and autoantibodies returning to normal three months after discontinuation of the medication (115). Currently, there are no case reports of risankizumab or guselkumab causing thyroid autoimmunity.

### 4.4 IL-17 inhibitors

The approved IL-17 pathway inhibitors interrupt the IL-23–Th17–IL-17 axis that drives psoriatic inflammation at different points. Secukinumab is a fully human IgG1κ monoclonal antibody that selectively neutralizes IL-17A, blocking its binding to IL-17RA. Ixekizumab, a similar drug, is a humanized IgG4 monoclonal antibody that also targets IL-17A, but has a higher affinity, resulting in a faster onset of action (61). Bimekizumab’s unique feature is its ability to target both IL-17A and IL-17F simultaneously (116). Brodalumab blocks the IL-17 receptor subunit A (IL-17RA), thereby interrupting the signaling of multiple IL-17 family members including IL-17A, IL-17F, IL-17C, IL-17A/F heterodimer, and IL-25, offering the broadest downstream signal interruption among currently approved IL-17 pathway inhibitors (116).

The onset of HT involves both the Th1 and Th17 pro-inflammatory pathways. Unlike dupilumab, which indirectly promotes Th1 by inhibiting Th2, the IL-17 inhibitor directly targets the Th17 pathway. In a mouse model of thyroiditis associated with ICIs, multiple lineages of IL-17A-producing T cells were found to be present in large numbers in thyroid tissue (117). Treatment with an anti-IL-17A antibody significantly reduced autoimmune infiltration of the thyroid tissue without affecting anti-tumor immune efficacy (117). Together with previous evidence implicating Th17 cells in the pathogenesis of HT, these findings suggest that the IL-17A axis may represent a potential therapeutic target for thyroid autoimmunity. However, this conclusion is based primarily on preclinical evidence and requires further validation in clinical studies. In the microenvironment of thyroid inflammation, IL-17F activates the recruitment of neutrophils and the NF-κB pathway (118, 119). Bimekizumab blocks IL-17F, which provides a more complete blockade of Th17 effects in theory (118, 120, 121). Its protective effect against HT may be stronger than that of the two-drug regimen that only blocks IL-17A (57, 118). However, it should be emphasized that this is still speculative and there are no specific clinical data on the effect of bimekizumab on HT.

However, the effectiveness of IL-17 inhibitors may differ depending on the organ and the inflammatory condition. In the thyroid, IL-17A-mediated Th17 cell infiltration is the mechanism of injury in HT, so blocking this process is beneficial. Meanwhile, the overall immune compensation still needs to be monitored. For instance, IL-17 can directly inhibit the differentiation of Th1 cells. Thus, when the IL-17 signal is absent, Th1 cells can induce severe colitis (122).

From the perspective of the immune mechanism, none of these three IL-17 inhibitors pose a theoretical risk of inducing or exacerbating HT in patients with simple psoriasis or psoriasis combined with HT. In fact, they may even have a mild beneficial effect on local Th17-mediated inflammation in the thyroid. Nevertheless, thyroid function should still be routinely monitored, as individual immune responses vary and there is currently a lack of large-sample clinical validation data.

### 4.5 JAK inhibitors

JAK-STAT signaling is a broadly pleiotropic intracellular pathway that transduces signals from a diverse range of cytokines, growth factors, and interferons (123). Beyond regulating inflammatory gene expression, it governs cell proliferation, differentiation, survival, hematopoiesis, and immune cell development (63). JAK inhibitors interrupt this intracellular signaling cascade, which serves as a shared downstream conduit for multiple cytokine receptors, including those for IL-4, IL-13, IL-5, IL-12, IL-31, IL-23, IL-17, thymic stromal lymphopoietin and interferon gamma (124).

The JAK kinases associated with the receptors undergo transphosphorylation to become activated, which is followed by the phosphorylation of STAT proteins. Phosphorylated pSTAT then forms homodimers or heterodimers and enters the nucleus to regulate the transcription of target genes. The JAK family comprises JAK1, JAK2, JAK3 and TYK2. The diversity of cytokine signaling is given by different combinations of the seven STAT proteins (125). JAK inhibitors prevent the phosphorylation activation of JAK by competitively occupying the ATP-binding pocket in the JAK kinase domain. This blocks the entire signaling cascade, preventing STAT from entering the cell nucleus and driving the transcription of inflammatory genes (126). Oral JAK inhibitors can be used simultaneously for the treatment of AD and AA.

Bioinformatics analysis of HT tissue confirmed that the IL-6/JAK/STAT3 pathway, IFN-γ signaling, IFN-α signaling and the inflammatory response pathway are key to the occurrence and development of HT (127). Overactivation of STAT3 may increase susceptibility to HT and elevate the titer of thyroid autoantibodies (127). In HT, CD8^+^ cytotoxic T lymphocytes dissolve thyroid cells directly through perforin and granzyme. The recruitment and activation of these cells depends on CD4^+^ T cells completing the process through IL-12 and IFN-γ signaling (2).

The IFN-γ receptor is coupled to JAK1 and JAK2 and primarily activates STAT1 downstream (128). The IFN-α receptor is coupled to TYK2 and JAK1 and signals downstream through formation of the STAT1, STAT2, and IRF9 complex known as ISGF3 (129). Classical and trans IL-6 signaling is transmitted through gp130, which can associate with JAK1, JAK2, and TYK2, and primarily activates STAT3 downstream (130). Therefore, JAK inhibitors that simultaneously inhibit both JAK1 and JAK2 should be particularly emphasized. Simultaneously blocking JAK1/2 inhibits both the IFN-γ-JAK1/2-STAT1 pathway, reducing abnormal thyroid MHC-II expression, apoptosis sensitivity, and the IL-6-JAK1/2-STAT3 pathway, reducing Th17 expansion and autoantibody production (131).

Interestingly, another study has found that, following the discontinuation of JAK inhibitors, a transient pro-inflammatory cascade reaction occurs. When the drugs are present, JAK undergoes accumulation of phosphorylation. Once the drugs are no longer present, the pre-activated pJAK become de-inhibited, which rapidly triggers a surge in pSTAT signaling and increases the expression of IFN and urokinase (132). It should be noted that this rebound mechanism was originally described in the context of major adverse cardiovascular events following JAK inhibitor withdrawal rather than in thyroid-specific studies (132). Its extrapolation to thyroid autoimmunity is a mechanistic inference that remains to be validated in dedicated clinical studies.

## 5 Discussion

### 5.1 The shared immunopathological foundation

The co-occurrence of AITDs with immune-mediated dermatological diseases, including vitiligo, CSU, AD, psoriasis and AA, reflects a shared immunological architecture rather than epidemiological coincidence. The pathological immune networks in AITDs and in these skin diseases overlap, it follows logically that some therapeutic interventions targeting these networks in the skin will also intersect, for better or for worse, with thyroid immune regulation.

### 5.2 The central paradox: how targeted immunotherapies can induce thyrotoxicosis

The most clinically counterintuitive and immunologically important finding of this review is the paradox that biologics with potent anti-inflammatory activity in their primary dermatological indication can paradoxically trigger or worsen thyroid inflammation, including thyrotoxicosis. This paradox is not a pharmacological anomaly. It is a predictable consequence of the bidirectional and interconnected nature of the immune system’s cytokine network.

Dupilumab exemplifies this paradox most clearly. As a potent Th2 suppressor, dupilumab is rigorously anti-inflammatory in AD, where Th2 excess is pathological. Yet by suppressing Th2, it removes the counter-regulatory restraint on Th1 polarization, thereby amplifying the Th1 dominance which drives thyroid destruction in HT. In addition, a case of dupilumab-induced thyrotoxicosis, manifesting as either new-onset GD have been reported (80). The induction of GD by a Th2-suppressing agent is the paradigmatic expression of this paradox. GD is conventionally classified as a Th2-mediated antibody disease, so its apparent induction by a Th2 inhibitor seems self-contradictory. Resolution requires recognizing that GD’s induction phase is Th1-driven. The pathogenic TRAb responsible for thyroid stimulation in GD belong predominantly to the IgG1 subclass, which is driven by Th1 cytokines, not Th2 (73). The Th2 dominance associated with GD characterizes the remission phase, not the initiation phase. Thus, dupilumab’s Th1-amplifying immune drift creates the ideal immunological conditions for GD induction, because of its anti-Th2 mechanism.

Ustekinumab presents a second, mechanistically distinct expression of the same paradox. By simultaneously blocking IL-12 (Th1-driving) and IL-23 (Th17-driving) via the shared p40 subunit, ustekinumab is effectively anti-inflammatory in psoriasis. However, by suppressing Th1, it removes Th1-mediated suppression of Th2 responses, permitting relative Th2 upregulation. The consequent increase in B cell activity and IgG autoantibody production may then create conditions permissive for antibody-mediated AITDs, including GD. A clinical case of autoimmune thyroiditis following ustekinumab, resolving after drug discontinuation, has been documented (115). Risankizumab and guselkumab, by sparing IL-12 and targeting only IL-23, avoid this Th2-rebound mechanism and represent a safer alternative in patients with AITDs risk.

Predicting which patients will experience this paradox requires an understanding of their baseline immune phenotype, including thyroid autoantibody status, and genetic polymorphisms in immune regulatory genes such as CTLA-4 and PTPN22.

### 5.3 Comparison of thyroid risks among five inhibitors

For patients with a family history of thyroid disease or pre-existing thyroid disorders, baseline thyroid screening should be performed before the initiation of ICIs. Thyroid function should also be monitored during treatment, given the well-established association between ICIs and thyroid immune-related adverse events. In contrast, thyroid dysfunction associated with dupilumab or ustekinumab has only been described in case reports. For patients at high risk of thyroid disease, periodic thyroid monitoring may therefore be considered (**Table 2**).

**Table 2:** The thyroid risk mechanisms and evidence levels of five targeted immunosuppressive agents.

Biologic class	Skin indication	Cytokine target	Mechanism	HT risk/benefit	Evidence level
IL-4Rα inhibitor	AD, CSU	IL-4/IL-13	Th2↓ → Th1↑ (drift)	Risk: HT/GD	Case report
IL-17 inhibitor	Psoriasis	IL-17A; IL-17A+F	Th17↓ → mild Th2 drift	Potential benefit; Th17-mediated damage ↓	Animal experiment
JAK inhibitor	AD, AA	JAK1/2; JAK1	IFN-γ-JAK1/2-STAT1↓ IL-6-JAK1/2-STAT3↓	Potential benefit	Mechanism speculation
IL-23/IL-12 inhibitor	Psoriasis	p40 (IL-12 + 23); p19 (IL-23)	Th17↓; Th1/Th2 rebalance (ustekinumab)	Risk: Th2 compensation → GD (ustekinumab)	Case report
Immune checkpoint inhibitor	Melanoma	CTLA-4 PD-1 PD-L1	Abnormal activation of autoreactive T cells	High risk: thyroiditis, HT,GD, Hypothyroidism, hyperthyroidism	Meta analysis Retrospective analysis Case report

↓ means a decrease, ↑ means an increase, → means that A leads to the occurrence of B.

To date, there have been no clear clinical reports linking JAK inhibitors or IL-17 inhibitors to the onset of HT or GD. Based on their known mechanisms, these agents do not appear to confer a significant risk of inducing AITDs. However, this conclusion should be interpreted with caution. The potential thyroid effects of JAK inhibitors are mainly inferred from mechanistic considerations. They remain insufficiently supported by animal studies or clinical trials. IL-17 inhibitors target the Th17 axis. Existing animal data suggest that anti-IL-17A therapy may reduce autoimmune thyroid infiltration (132). However, any potential protective effect requires further validation in clinical studies.

### 5.4 Limitations

This review is a narrative synthesis, which is dominated by mechanistic studies and case reports rather than prospective cohort data. The causal inferences drawn from case reports must be interpreted with caution, as thyroid autoimmunity events may be coincidental to rather than caused by targeted immunotherapies therapy in the dermatological patient population. Furthermore, the long-term thyroid safety data for most of these agents remain immature, and individual variability in immunological response limits the generalizability of mechanistic predictions to individual patients.

## 6 Conclusion

The five classes of targeted immunotherapies may have different implications for HT and GD because of their distinct mechanisms of action. JAK inhibitors and IL-17 inhibitors may theoretically exert neutral or even protective effects on thyroid autoimmunity. In contrast, other biologic agents have been associated with varying degrees of potential thyroid-related risk. However, the strength of evidence differs substantially among drug classes.

Among these therapies, systematic thyroid monitoring before and during treatment is most clearly warranted for ICIs. ICIs are currently the drug class with the strongest evidence supporting thyroid function assessment at baseline and during treatment. Monitoring usually includes TSH and free T4.

Thyroid-related events associated with dupilumab and ustekinumab have so far been reported mainly in isolated case reports. Therefore, routine thyroid screening should not be regarded as mandatory for all patients receiving these agents. However, thyroid monitoring may be considered in selected high-risk individuals. These include patients with a previous history of thyroid disease, positive thyroid autoantibodies, a family history of thyroid disease, or coexistence of multiple autoimmune diseases.

For such high-risk patients, TSH and FT4 may be added to the routine assessment before or during treatment. TPOAb and TgAb may also be considered when autoimmune thyroid disease is suspected. TRAb testing may be reserved for patients with clinical or biochemical features suggestive of GD.
